# Assistive diagnostic technology for congenital heart disease based on fusion features and deep learning

**DOI:** 10.3389/fphys.2023.1310434

**Published:** 2023-11-23

**Authors:** Yuanlin Wang, Xuankai Yang, Xiaozhao Qian, Weilian Wang, Tao Guo

**Affiliations:** ^1^ School of Information Science and Engineering, Yunnan University, Kunming, China; ^2^ Research Center for Complexity Sciences, Hangzhou Normal University, Hangzhou, China; ^3^ Fuwai Cardiovascular Hospital of Yunnan Province, Kunming, China

**Keywords:** heart sounds, congenital heart disease, fusion features, attention mechanism, network

## Abstract

**Introduction:** Congenital heart disease (CHD) is a cardiovascular disorder caused by structural defects in the heart. Early screening holds significant importance for the effective treatment of this condition. Heart sound analysis is commonly employed to assist in the diagnosis of CHD. However, there is currently a lack of an efficient automated model for heart sound classification, which could potentially replace the manual process of auscultation.

**Methods:** This study introduces an innovative and efficient screening and classification model, combining a locally concatenated fusion approach with a convolutional neural network based on coordinate attention (LCACNN). In this model, Mel-frequency spectral coefficients (MFSC) and envelope features are locally fused and employed as input to the LCACNN network. This model automatically analyzes feature map energy information, eliminating the need for denoising processes.

**Discussion:** The proposed classification model in this study demonstrates a robust capability for identifying congenital heart disease, potentially substituting manual auscultation to facilitate the detection of patients in remote areas.

**Results:** This study introduces an innovative and efficient screening and classification model, combining a locally concatenated fusion approach with a convolutional neural network based on coordinate attention (LCACNN). In this model, Mel-frequency spectral coefficients (MFSC) and envelope features are locally fused and employed as input to the LCACNN network. This model automatically analyzes feature map energy information, eliminating the need for denoising processes. To assess the performance of the classification model, comparative ablation experiments were conducted, achieving classification accuracies of 91.78% and 94.79% on the PhysioNet and HS databases, respectively. These results significantly outperformed alternative classification models.

## 1 Introduction

Congenital Heart Disease (CHD) is a group of severe congenital anomalies that profoundly affect the physical health of adolescents. Without timely medical intervention, these conditions can significantly impair quality of life and even lead to mortality. Consequently, early screening plays a crucial role in enhancing patient survival rates and overall wellbeing (Hoffman et al., 2004). Decades of clinical experience have demonstrated that Phonocardiogram (PCG) holds vital physiological and pathological information about the heart, serving as a pivotal diagnostic basis for cardiovascular diseases (Rangayyan and Lehner, 1987). The first heart sound (S1) and the second heart sound (S2) constitute primary components of heart sounds and are of paramount clinical interest. In the past, patients residing in remote areas faced high medical costs and limited healthcare resources. Disease screenings were predominantly carried out through medical teams dispatched to local hospitals, incurring substantial personnel, material, and financial resources. Manual auscultation demanded doctors possess extensive auscultatory expertise, yet yielded low detection rates and lacked means to preserve auscultation data. Consequently, researchers worldwide have long been engaged in the exploration of automated heart sound diagnostics, initiating diverse avenues of research. For instance, Zabihi et al. (2016), in 2016, employed the PhysioNet Challenge database for Cardiology, selecting 18 feature subsets from the time domain, frequency domain, and time-frequency domain (such as wavelet transform, Mel-Frequency Cepstral Coefficients - MFCC, etc.). Their approach achieved an accuracy of 85.90% on the test set. Tan et al. (2019). adopted MFCC for time-frequency analysis of PCG signals, constructing feature maps from spectral coefficients obtained via Mel filters. They proposed an automated model for coronary artery disease classification. Chen et al (Chen and Zhang, 2020). Employed Short-Time Fourier Transform (STFT) spectrograms as input to a Convolutional Neural Network (CNN), achieving an accuracy of 95.49% on 39 test samples. Rubin et al. (2016). Selected 3,240 heart sounds from the heart sound challenge dataset and employed MFCC combined with CNN to realize heart sound classification, attaining an accuracy of 84.80% on the test set.

We propose a novel classification model of locally superimposed fusion features and LCACNN with attention mechanism. By comparing with recent research in this field, the main contributions of this article are as follows:

(1) Uniqueness of Database: To the best of our knowledge, this paper employs the first database specifically curated for congenital heart diseases. Zeinali et al.(Zeinali and Niaki, 2022) and Ren et al. (2022) only used the PhysioNet database for heart sound classification experiments. However, the PhysioNet data set has a small amount of data, a large age gap between patients, and poor data quality (because the challenge is in a cluttered environment, the heart sound collection process is interference).
(2) Superior Performance: Across various performance metrics, our classification model significantly outperforms other comparative and ablation methods Chen et al. (2022). Employed the CNN-LSTM network to classify heart disease and achieved an accuracy of 85%. Rath et al. (2022) used machine learning methods to detect heart sounds and obtained an accuracy of 85.08%. In addition, the larger volume of heart sound data we used makes the results more convincing.
(3) Enhanced Screening in Remote Areas: In remote screening settings, the proposed classification model demonstrates a strong capability to replace auscultation specialists, eliminating the need for specialized auscultation training.

In the Materials and Methods section, we describe the division of cardiac cycles and heart sound segmentation methods, and extract and fuse the MFSC and homomorphic envelope features of the segmented heart sounds. In the classification model we build LCACNN. In the results section, we conduct comparative experiments on the HS database and PhysioNet database to verify our method. In the discussion and conclusion sections, we analyze the role of fusion features and attention mechanisms, and draw the reasons for the effectiveness of our method based on experimental results.

## 2 Materials and methods

### 2.1 Description of experimental data

The experimental data in this study originated from two datasets: (1) A cardiac sound database created from samples collected by our research group at Yunnan Fuwai Cardiovascular Hospital and during congenital heart disease screenings in various mountainous primary schools across Yunnan province. The age range of the cardiac sound volunteers was between 8 months and 18 years. The HS database was recorded using The ONE ThinkLabs electronic stethoscope, with a sampling frequency of 5,000 Hz and a recording duration of 20 s. The database consists of 133 synchronized heart sound-electrocardiogram recordings (HS_ECG database) and 7,000 heart sound recordings (HS database). Abnormal patient samples obtained during the screening process were subsequently confirmed by following ultrasound examinations and hospital diagnosis. These abnormal cases encompassed common congenital heart disease types, including Atrial Septal Defect (ASD), Ventricular Septal Defect (VSD), and Patent Ductus Arteriosus (PDA). The distribution of positive and negative samples was balanced. (2)The PhysioNet database from the 2016 PhysioNet/CinC Challenge (Springer et al., 2016), containing 3,240 heart sound recordings. The ratio of normal volunteer heart sound recordings to abnormal volunteer heart sound recordings was 4:1. The sampling rate was 2,000 Hz, and the recording duration varied from 5 to 120 s. The PhysioNet database was divided into training set (70%), testing set (20%), and validation set (10%).

### 2.2 Experimental procedure

**FIGURE 1:**
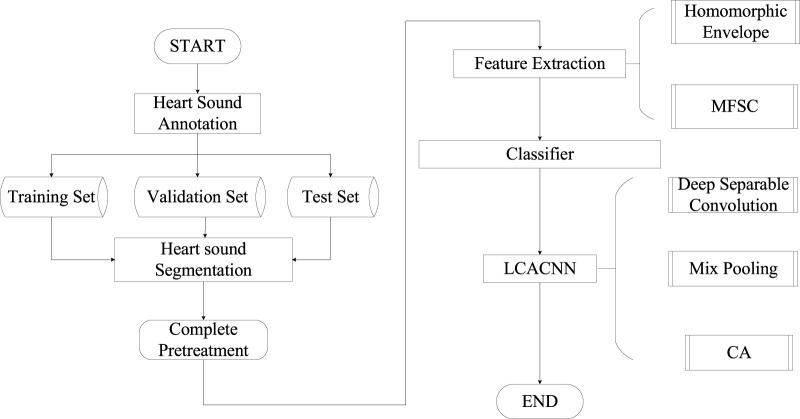
Procedure of CHD classification model.

The classification model proposed in this study involves the following steps, as illustrated in Figure 1: (1) Initially, cardiac sound signals are annotated based on electrocardiogram (ECG) signals to establish a baseline. Subsequently, using the duration of cardiac cycles as a constraint, a Hidden Markov Model (HMM) is employed to model the cardiac sound signals, resulting in segmented and localized cardiac sound signals. (2) The segmented cardiac sound signals undergo feature extraction, where considering the primary components of S1 and S2, a fusion of Homomorphic Envelope Features and Mel-Frequency Spectral Coefficients (MFSC) is introduced for local feature integration. (3) The feature maps are fed into the LCACNN network. To enhance focus on the S1 and S2 regions, a Channel Attention (CA) mechanism is incorporated. To meet practical screening requirements, Depthwise Separable Convolutions (DC) are employed instead of standard convolution modules to reduce model parameter count. Mixed Pooling (MP) is used in the pooling layers as a replacement for both Max Pooling and Average Pooling, alleviating issues related to granularity of information aggregation to some extent.

### 2.3 Heart sound segmentation

In the heart sound segmentation model, the R-wave and T-wave in the electrocardiogram correspond to the S1 and S2 periods of the heart sound signal (Zhu et al., 2018). The peak of the R-wave corresponds to the start of the S1 period, while the end of the T-wave corresponds to the start of the S2 period (Manikandan and Soman, 2012). The S1, S2, systolic, and diastolic periods need to be labeled based on the R-peak and T-wave (Hempel et al.) The average time ( $\bar{S}1\pm \sigma_{S1}$ ) between the R peak and the next R peak is set as S1. The peak value of the heart sound signal S2 corresponding to the end of the T wave is the largest. This peak is used as the S2 average point, so S2 can be recorded as the average time around the peak $\left(\frac{\left(\bar{S}2\pm \sigma_{S2}\right)}{2}\right)$ . The systole is between S1 and S2, and the diastole is between S2 and the next S1.

Heart sound can be regarded as a quasi-periodic, short-term stationary signal, with slight differences between each cycle. The autocorrelation method can be used to analyze the heart sound signal to obtain a complete cardiac cycle. We use the autocorrelation of the Hilbert envelope to extract the cardiac cycle (Qin and Zhong, 2006), and the autocorrelation coefficient is shown in Eq 1. N is the total sampling length of the heart sound signal, and ${\tilde{r}}_{x}\left(l\right)$ is its autocorrelation coefficient.

$${\tilde{r}}_{x}\left(l\right)=\frac{1}{N}\sum_{n=0}^{N-l-1}x\left(n+l\right)x\left(n\right)$$ (1)

The traditional Hidden Markov Models (HMM) partition the four phases of heart sounds with equal probabilities (Yin et al., 2022). However, the four periods in which the human body actually produces heart sounds have unequal durations. Using HMM for segmentation can easily lead to segmentation errors in S1, systole, S2, and diastole (Tokuda et al., 2002). Therefore, we introduce duration into the HMM model to further approximate the heart sound period pattern in reality. We define the duration probability constraint function ${p_{j}}^{d}$ , which represents the occurrence probability of constraint duration d in a certain j period, $j\in \left\{C_{1},C_{2},C_{3},C_{4}\right\}$ . A is the period probability state transition matrix. It is known from clinical experience that the four phases of heart sound always appear in a specific pattern, namely, S1, systole, S2 and diastole, so A can be written as Eq 2.

$$A=\left(\begin{array}{cccc} 0 & 1 & 0 & 0\\ 0 & 0 & 1 & 0\\ 0 & 0 & 0 & 1\\ 1 & 0 & 0 & 0 \end{array}\right)$$ (2)

Since there are only four heart sound status periods i=1,2,3,4 . In the actual collection process of heart sounds, the time when we place the auscultation probe may correspond to any period of the cardiac cycle, so the initial state probability π is shown in Eq 3.

$$\pi =P\left\{c_{t}=C_{i}\right\}=1/4$$ (3)

**FIGURE 2:**
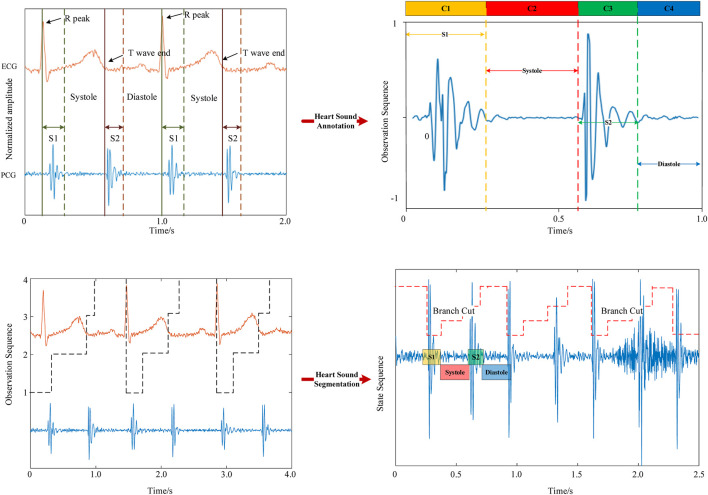
Heart sound segmentation results.

Then, we need to perform DHMM modeling on the heart sounds, and the average value $d\mu_{j}$ and variance d∑j of the duration are used to measure the model parameters λ . The observation formula is shown in Eq 4. Finally, the Viterbi algorithm is employed to perform model decoding (Guo et al., 2022), completing the heart sound segmentation process, as shown in Figure 2.

$$\lambda =\left\{a_{ij},\pi_{i},{p_{j}}^{d},d\mu_{j},d\sum j\right\}$$ (4)

### 2.4 Fusion features

Time-frequency domain features are widely employed in heart sound analysis, encompassing both temporal and spectral characteristics (You et al., 2022). These features effectively capture the relationship between frequency and energy. Mel-frequency spectral coefficients (MFSC) are a commonly used method for extracting time-frequency domain features. For instance, Li et al. (2022a) differentiated between normal and pathological heart sounds using MFSC features. Meanwhile, the Homomorphic Envelope (Monteiro et al., 2022), as a morphological feature, reflects changes in the waveform of heart sounds. To accentuate the differences between normal and congenital heart disease (CHD) heart sounds in the S1 and S2 components, this study considers the joint use of temporal envelope features and MFSC features for feature analysis.

The process for obtaining MFSC features involves the following steps: Fast Fourier Transform (FFT) is often used to transform signals from the time domain to the frequency domain(Li et al., 2022b), which can reflect the energy changes of the signal, as shown in Eq 5. The heart sound signal is a weak signal and its energy spectrum cannot be observed in the time domain. Therefore, the time domain characteristics of the heart sound can be converted into frequency domain information through FFT.

$$X\left(k\right)=\sum_{n=0}^{N-1}x\left(n\right)e^{\frac{j2\pi kn}{N}},0\leq n,k\leq N-1$$ (5)

Firstly, the heart sound signal undergoing frame processing is subjected to FFT, followed by the calculation of energy values A in corresponding frequency bands using a Mel filter bank. We define the Mel filter group $H_{n}\left(k\right)$ to contain N Mel scale filters, where $H_{n}\left(k\right)$ is shown in Eq 6.

$$H_{n}\left(k\right)=\left\{\begin{array}{l} 0,k<f\left(n-1\right)\\ \frac{k-f\left(n-1\right)}{f\left(n\right)-f\left(n-1\right)},f\left(n-1\right)\leq k\leq f\left(n\right)\\ 1,k=f\left(n\right)\\ \frac{f\left(n+1\right)-k}{f\left(n+1\right)-f\left(n\right)},f\left(n\right)<k\leq f\left(n+1\right)\\ 0,k>f\left(n+1\right) \end{array}\right.$$ (6)

We calculate the obtained $X\left(k\right)$ and $H_{n}\left(k\right)$ through each Mel filter to get the output logarithmic energy $s\left(n\right)$ , and the unit of physical quantity is db . Finally, we convert PCG from the time domain to the time-frequency domain for analysis. This process yields the MFSC features, as depicted in Eq 7.

$$s\left(n\right)=\ln\left(\sum_{k=0}^{M-1}{\left|X\left(k\right)\right|}^{2}H_{n}\left(k\right)\right),0\leq n\leq N$$ (7)

Here, $X\left(k\right)$ represents the transformed frequency-domain heart sound signal, N stands for the number of signal samples in each frame, $H_{n}\left(k\right)$ represents the frequency response of the Mel filter bank, and n denotes the Mel scale filter in the current filter bank, and the units of physical quantities are HZ/db , F is the center frequency of the nth Mel scale filter, and the unit of physical quantity is HZ .

The computation of the Homomorphic Envelope involves three steps: Firstly, a Butterworth filter is used to filter the signal (Siew et al., 2022), resulting in a transfer function. Subsequently, the preprocessed heart sound signal undergoes Hilbert transformation, producing its narrow-band signal. Finally, the narrow-band signal is passed through a zero-phase filter to obtain the envelope of the heart sound signal, as depicted in Eq 8.

$$H_{e}=\exp\left[\log a\left(t\right)\right]$$ (8)

Here, $a\left(t\right)$ represents the low-frequency component of the signal, and $H_{e}$ represents the homomorphic envelope signal.

The envelope signal obtained from the previous step is partitioned, with individual cardiac cycles of the same volunteer at the same position treated as a reference envelope. This reference envelope then undergoes maximum pooling, where the maximum value is selected within a defined sampling region. The sampling region should not be excessively large, as doing so might lead to the loss of pathological information. In this study, the minimum sampling region comprises three adjacent sample points. The sampled reference envelope, denoted as $H_{e}^{\prime }$ , subsequently undergoes a non-linear processing step, as illustrated in Eq 9.

$$Q_{i}\left(t\right)=\log\left[H_{e}^{\prime }\right]$$ (9)

Subsequently, multiple non-linearized reference envelopes $Q_{i}\left(t\right)$ are superimposed to form a two-dimensional matrix Q . The matrix Q undergoes centralization and amplitude normalization to yield G , as described by Eq 10.

$$G=\frac{Q-\bar{Q}}{\max\left(\left|Q-\bar{Q}\right|\right)}$$ (10)

**FIGURE 3:**
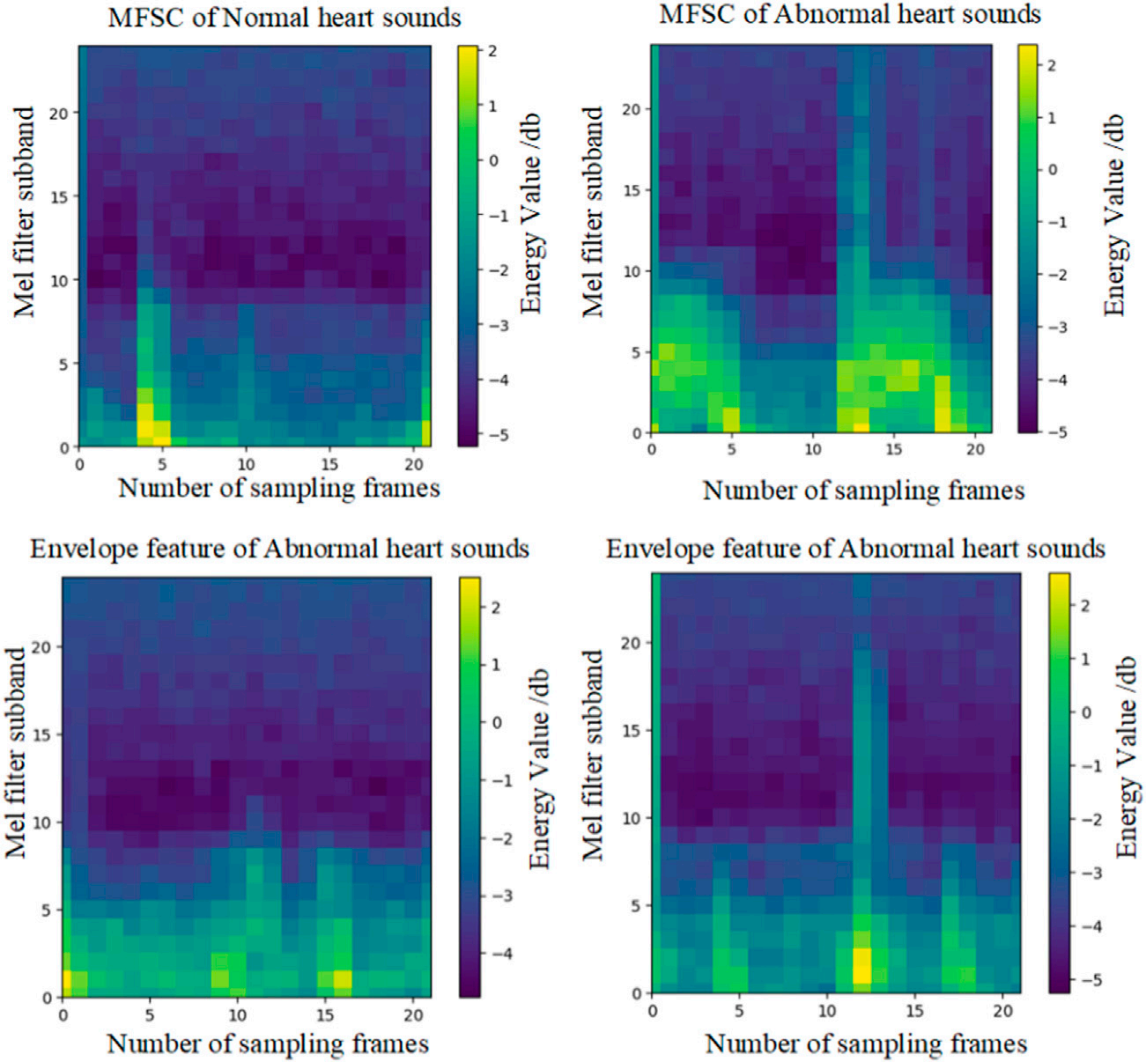
Envelopes and MFSC feature maps of normal and CHD heart sounds.

Finally, energy value encoding visualization is performed on C. Locations with higher energy values are assigned a brighter color, while locations with lower energy values are assigned a darker shade. The hue values selected for shading are consistent with the energy hues of MFSC features, as illustrated in Figure 3.

In feature engineering, feature fusion refers to the process of combining two distinct categories of features in a manner that enhances their performance capabilities (Xiang et al., 2023). To prevent the generation of excessive redundant information due to fusion, and considering the research objectives and the analysis of pathological features, this study proposes a local overlay fusion strategy.

The local overlay fusion strategy involves the following steps: Firstly, in the analysis of pathological features, a cardiac cycle is approximately 0.8 s long, with the S1 and S2 phases each lasting around 0.2 s. Since heightened S1 and S2 phases result in elevated energy values, a sliding window (Hou et al., 2022) is set to detect energy value magnitudes within the 0.2-s interval. If the energy values of the sampled frames within the window exceed a predetermined threshold (derived as the average energy value of an individual cardiac cycle in this study), the phase is identified as an elevated energy period. The subsequent process is exemplified using the S2 phase.

The MFSC feature matrix corresponding to the S2 phase is overlaid with the energy values of the envelope feature matrix corresponding to the S2 phase. For the remaining regions, the average is taken, as depicted in Eqs 11, 12.

$$W\_S_{2}=M\_S_{2}+G\_S_{2}$$ (11)

$$W_{o}=\frac{M_{o}+G_{o}}{2}$$ (12)

Here, $W_{o}$ , $M_{o}$ , and $G_{o}$ represent the average energy values for the remaining periods, corresponding to the energy values of the remaining periods in matrix M and the energy values of the remaining periods in matrix.

**FIGURE 4:**
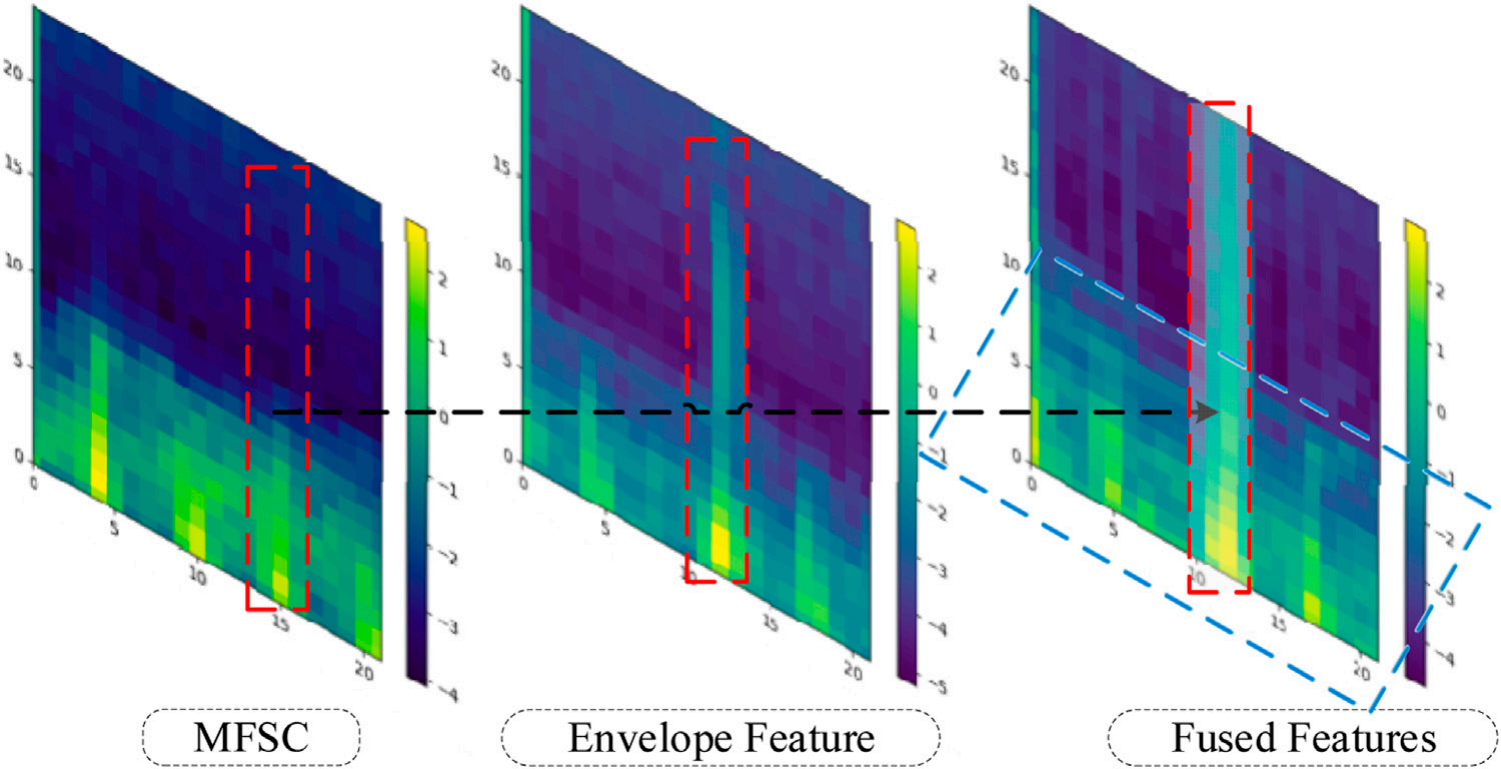
Local overlay.

Finally, energy value encoding is performed to generate the illustrative diagram of the fused feature, as depicted in Figure 4. The red shaded area corresponds to the energy value overlay region, while the blue shaded area corresponds to the energy value averaging region.

### 2.5 Classification network

In recent years, neural networks have been widely applied in fields like heart sound analysis, achieving significant advancements in classification and recognition tasks. In the domain of heart sound classification, Xiao et al. employed CNN for sound classification (Xiao et al., 2020). CNN networks have demonstrated their efficacy in handling local details and feature maps in classification tasks (Liu et al., 2021). Therefore, this paper proposes an LCACNN classification network model.

The classification network comprises four sets of modules, each containing convolutional layers, mixed pooling layers, and CA attention mechanisms. The first two convolutional layers are standard convolutions, while the latter two layers employ depthwise separable convolutions. This combination of standard and depthwise separable convolution layers is chosen due to the input feature dimensionality and richness in detail in the initial layers, leading to the use of standard convolutions for the first two layers to improve classification accuracy. For the subsequent layers where spatial feature influence is reduced, depthwise separable convolutions are utilized to reduce parameter count.

The mixed pooling strategy combines the advantages of max pooling and average pooling (Yu et al., 2014), requiring no additional hyperparameter tuning and incurring minimal computational overhead. It to a certain extent addresses the issues of potential loss of local details and redundant information in heart sound feature maps associated with max and average pooling. This helps reduce the risk of overfitting. Let $y_{ij}^{k}$ represent the output value of sub-region $R_{ij}$ partitioned from the k th matrix, and let λ=0,1 denote a random value (with 1 representing max pooling operation and 0 representing average pooling operation). The calculation process is illustrated in Eqs 13, 14.

$$y_{ij}^{k}=\lambda \cdot {\hat{y}}_{ij}^{k}+\left(1-\lambda \right)\cdot {\bar{y}}_{ij}^{k}$$ (13)

$$y_{ij}^{k}=\lambda \cdot \max_{\left(p,q\right)\in R_{ij}}x_{pq}^{k}+\left(1-\lambda \right)\cdot \frac{1}{\left|R_{ij}\right|}\sum_{\left(p,q\right)\in R_{ij}}x_{pq}^{k}$$ (14)

**FIGURE 5:**
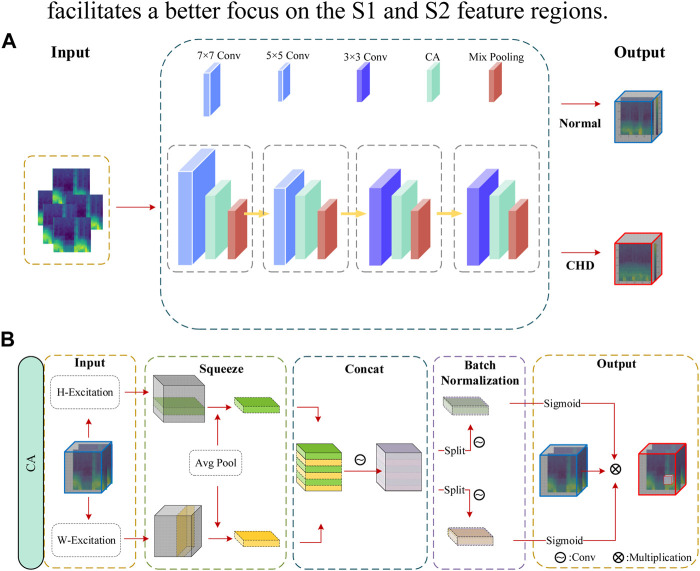
**(A)** LCACNN network architecture **(B)** design of the CA attention mechanism module.

In this study, a comparison was made among common attention mechanisms such as SE (Woo et al., 2018) and CBAM (Hou et al., 2021), and a coordinate-based attention mechanism was proposed. The principle of the CA attention mechanism is illustrated in Figure 5. Initially, the input feature block ( c×h×w ) undergoes global average pooling (GAP) for compression to obtain activations along the horizontal axis ( w ) and vertical axis ( h ), resulting in $M_{W}\left(\cdot \right)$ and $M_{H}\left(\cdot \right)$ respectively(Kumar et al., 2021). This embeds spatial information into channels at different positions. Subsequently, $M_{W}\left(\cdot \right)$ and $M_{H}\left(\cdot \right)$ are concatenated and processed through a convolutional module. After passing through a Batch Normalization (BN) layer, the concatenated feature block is split and subjected to separate convolution operations (Lyu et al., 2022). The weight values are obtained by applying the sigmoid activation function to the split features, which are then multiplied with the input feature block to yield an output feature block imbued with both spatial and channel information. This construction facilitates a better focus on the S1 and S2 feature regions.

## 3 Results

The signal preprocessing (heart sound segmentation), feature extraction (MFSC, envelope features), and construction of the deep learning model (LCACNN) in this research were executed on a system equipped with Ryzen 7 4800H @ 2.90GHz, NVIDIA GeForce RTX 2060 6GB, and 32 GB of memory. The deep learning framework employed was TensorFlow 2.0 from Google, utilizing the programming language Python 3.8.

### 3.1 Model construction experiment

The LCACNN model was configured with the following settings: Adam optimizer was employed with a learning rate set to 0.001. Sigmoid was chosen as the primary binary classification activation function, and Batch Normalization (BN) layers were introduced to mitigate model overfitting. The batch size for training was uniformly set at 64, and the model underwent 100 epochs of training. We used the binary_crossentropy loss function commonly used in binary classification problems (Mantas, 2023), which is often used to evaluate the effectiveness of neural network models in classifying tasks between two categories. The mathematical expression of the binary_crossentropy loss function is shown in Eq 15. $L\left(y,p\right)$ represents the binary_crossentropy loss function, y is the actual label (0 or 1), and p is the predicted probability of the model.

$$L\left(y,p\right)=-\left[y\cdot \log\left(p\right)+\left(1-y\right)\cdot \log\left(1-p\right)\right]$$ (15)

**FIGURE 6:**
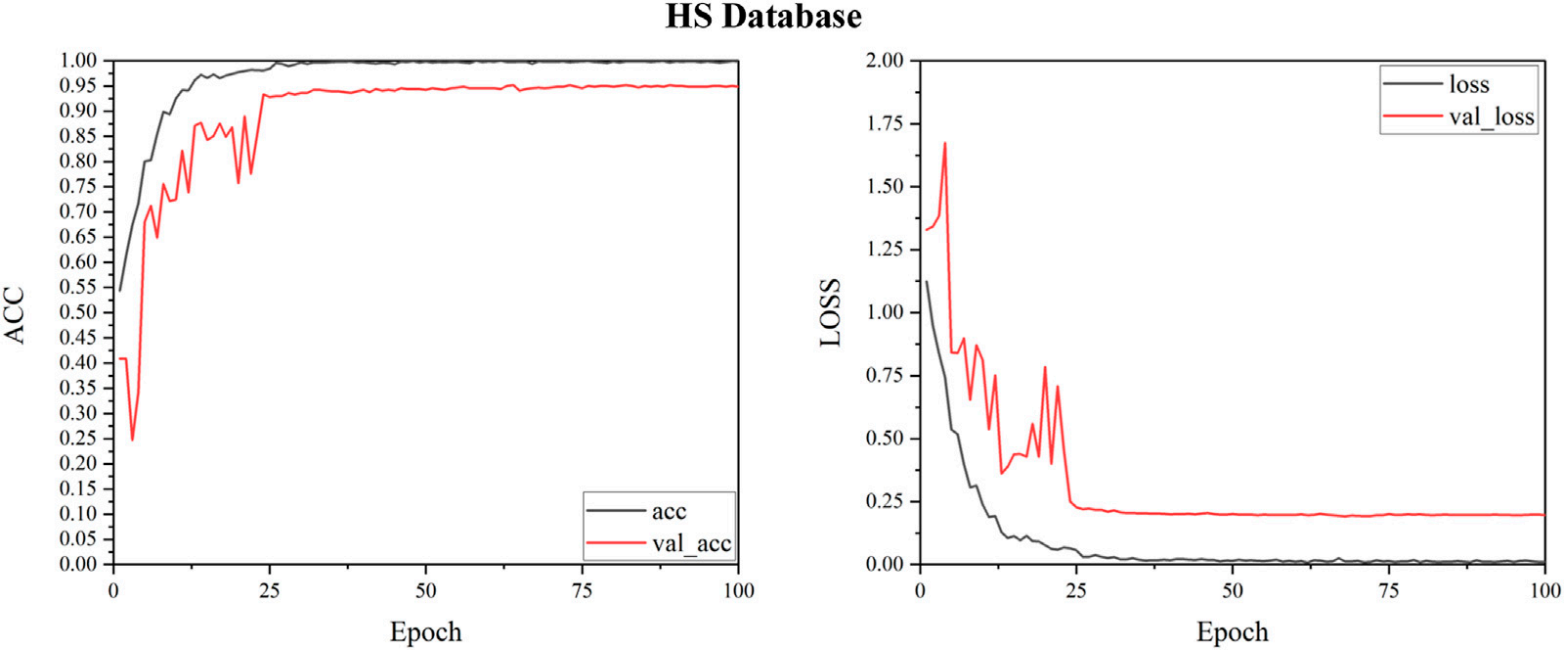
Loss function.

In order to explore the impact of the selected module group (Conv, CA and Pooling) on the experimental results, We utilized 4,900 heart sounds from the HS database for our training set and 700 heart sounds for the test set. According to the results in Table 1, we successfully built the network structure. The experimental results show that under the LCACNN network structure with 4 groups of modules, the accuracy reached the highest value and the loss value also reached the lowest point. We conducted model training on this network structure based on the HS database. The visualization results of the loss value during the training process are shown in Figure 6.

**TABLE 1:** Relationship between Acc and loss value under the module.

Module/piece	Acc	Loss
1	79.57	2.02
2	85.43	1.49
3	90.21	2.10
4	94.79	0.21
5	77.65	2.99

### 3.2 Performance evaluation experiment

The performance evaluation experiment consists of comparative experiments and ablation experiments (Meyes et al., 2019). Five evaluation metrics are employed to assess the model’s performance in CHD classification. These metrics include classification accuracy (Acc), classification sensitivity (Se), and classification specificity (Sp), as depicted in Equations (16)–(18).

$$S_{e}=\frac{\text{TP}}{\text{TP}+\text{FN}}\times 100\%$$ (16)

$$S_{p}=\frac{\text{TN}}{\text{TN}+\text{FP}}\times 100\%$$ (17)

$$\text{Acc}=\frac{\text{TP}+\text{TN}}{\text{TP}+\text{TN}+\text{FP}+\text{FN}}\times 100\%$$ (18)

Here, TP denotes the number of correctly identified anomalies, also known as true positives; FN represents the number of undetected anomalies, termed as false negatives; TN signifies the number of correctly identified normal cases, denoted as true negatives; FP accounts for the number of falsely identified normal cases, referred to as false positives.

The other two metrics are the Modified Accuracy (MAcc) and the F-Score ( $F_{\beta }$ ). The MAcc metric is introduced to account for the influence of external noise factors on accuracy. Following the evaluation guidelines of the PhysioNet Challenge, unweighted coefficients ( ∂ ) are incorporated into the specificity (Sp) and sensitivity (Se) metrics, and their values are set to 0.5 to derive the Modified Accuracy (MAcc) metric, as demonstrated in Eq 19.

$$\text{MAcc}=\frac{S_{e}+S_{p}}{2}$$ (19)

The risk of false negatives in clinical practice is significantly greater than that of false positives. False positives can be further excluded through subsequent examinations, whereas false negatives can delay treatment due to screening result inaccuracies. Thus, reducing false negative cases holds paramount importance in clinical settings. To address this, the present study introduces the $F_{\beta }$ index for further assessment, as shown in Eq 20, where $F_{\beta }$ represents the harmonic coefficient of both Sp and Se. In this research, we adopt the $F_{1}$ index to harmonize the Sp and Se coefficients of the binary classification model.

$$F_{\beta }=\left(1+\beta^{2}\right)\times \frac{S_{p}\cdot S_{e}}{\beta^{2}\cdot S_{p}+S_{e}}$$ (20)

Based on the aforementioned approach, comparative experiments between the feature extraction and classification algorithm proposed in this study and other algorithms are presented in Tables 2, 3. Ablation experiments are demonstrated in Tables 4, 5. Both the comparative experiments and our proposed algorithm were conducted on the same test dataset. In the PhysioNet database, the training set comprises 4,900 heart sounds, and the test set consists of 700 heart sounds. Regarding the HS database, the training set comprises 2,268 heart sounds, and the test set encompasses 324 heart sounds.

**TABLE 2:** Comparative experiments of various algorithms for CHD (PhysioNet database).

Classification algorithm	Heart sound classification evaluation Metrics(%)				
	Acc	S_e_	S_p_	MAcc	*F-score*
STFT + CNN	88.13	83.03	91.97	87.50	87.27
MFCC + CNN	89.33	84.41	92.37	88.39	88.21
MFSC + CNN	90.03	85.42	94.01	89.21	88.95
MFSC+ Envelope Features+ LCACNN	**91.78**	**90.43**	**92.47**	**91.45**	**91.43**

Bold indicates the resulting values.

**TABLE 3:** Comparative experiments of various algorithms for CHD (HS database).

Classification algorithm	Heart sound classification evaluation Metrics(%)				
	Acc	S_e_	S_p_	MAcc	*F-score*
STFT + CNN	88.61	84.11	92.37	88.24	88.04
MFCC + CNN	90.03	85.87	93.36	89.62	89.46
MFSC + CNN	91.11	86.04	95.31	90.68	90.44
MFSC+ Envelope Features+ LCACNN	**94.79**	**93.41**	**95.77**	**94.59**	**94.58**

Bold indicates the resulting values.

**TABLE 4:** Comparison of ablation experiments for CHD (PhysioNet database).

Classification algorithm	Heart sound classification evaluation Metrics(%)				
	Acc	S_e_	S_p_	MAcc	*F-score*
MFSC+ CNN	90.03	85.42	94.01	89.21	88.95
Envelope Features+ CNN	89.12	83.91	93.88	88.90	88.62
MFSC+ Envelope Features+ CNN	90.84	85.89	95.07	90.48	90.25
MFSC+ Envelope Features+ SE+ CNN	89.83	86.33	93.42	89.88	89.74
MFSC+ Envelope Features+ CBAM+ CNN	90.92	86.44	95.09	90.76	90.55
MFSC+ Envelope Features+ LCACNN	**91.78**	**90.43**	**92.47**	**91.45**	**91.43**

Bold indicates the resulting values.

**TABLE 5:** Comparison of ablation experiments for CHD (HS database).

Classification algorithm	Heart sound classification evaluation Metrics(%)				
	Acc	S_e_	S_p_	MAcc	*F-score*
MFSC + CNN	91.11	86.04	95.31	90.68	90.44
Envelope Features+ CNN	90.06	84.11	94.12	89.12	88.83
MFSC+ Envelope Features+ CNN	91.85	87.17	95.81	91.49	91.29
MFSC+ Envelope Features+ SE+ CNN	89.86	86.37	93.21	89.79	89.66
MFSC+ Envelope Features+ CBAM+ CNN	92.51	88.04	96.32	92.18	91.99
MFSC+ Envelope Features+ LCACNN	**94.79**	**93.41**	**95.77**	**94.59**	**94.58**

Bold indicates the resulting values.

## 4 Discussion

### 4.1 Analysis of fusion feature experiments

**FIGURE 7:**
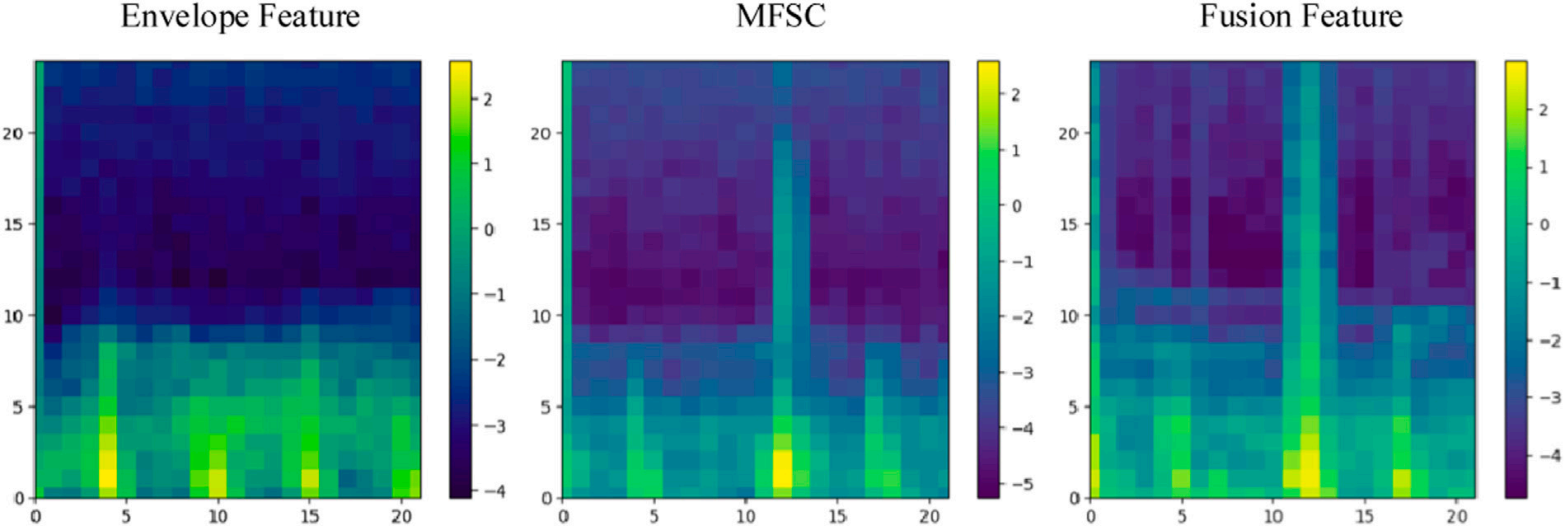
Envelope features, MFSC, and fusion feature.

The effectiveness of fusion features will be analyzed in this section, considering both experimental theory and results. A wealth of pathological information is contained within S1 and S2, and the primary intention behind the design of the feature extraction process is to accentuate the finer details of S1 and S2. As depicted in Figure 7, the amplitude information in the time domain of the Phonocardiogram (PCG) is mainly emphasized by envelope features, while Mel-frequency spectral coefficients (MFSC) concentrates on the representation of energy information in the time-frequency domain of the PCG. By combining the information from both sources, fusion features are capable of encompassing a more extensive range of detailed information. As evidenced by Tables 4, 5, superior performance is consistently exhibited by fusion features across all evaluation metrics.

### 4.2 Analysis of attention mechanism experiments

**FIGURE 8:**
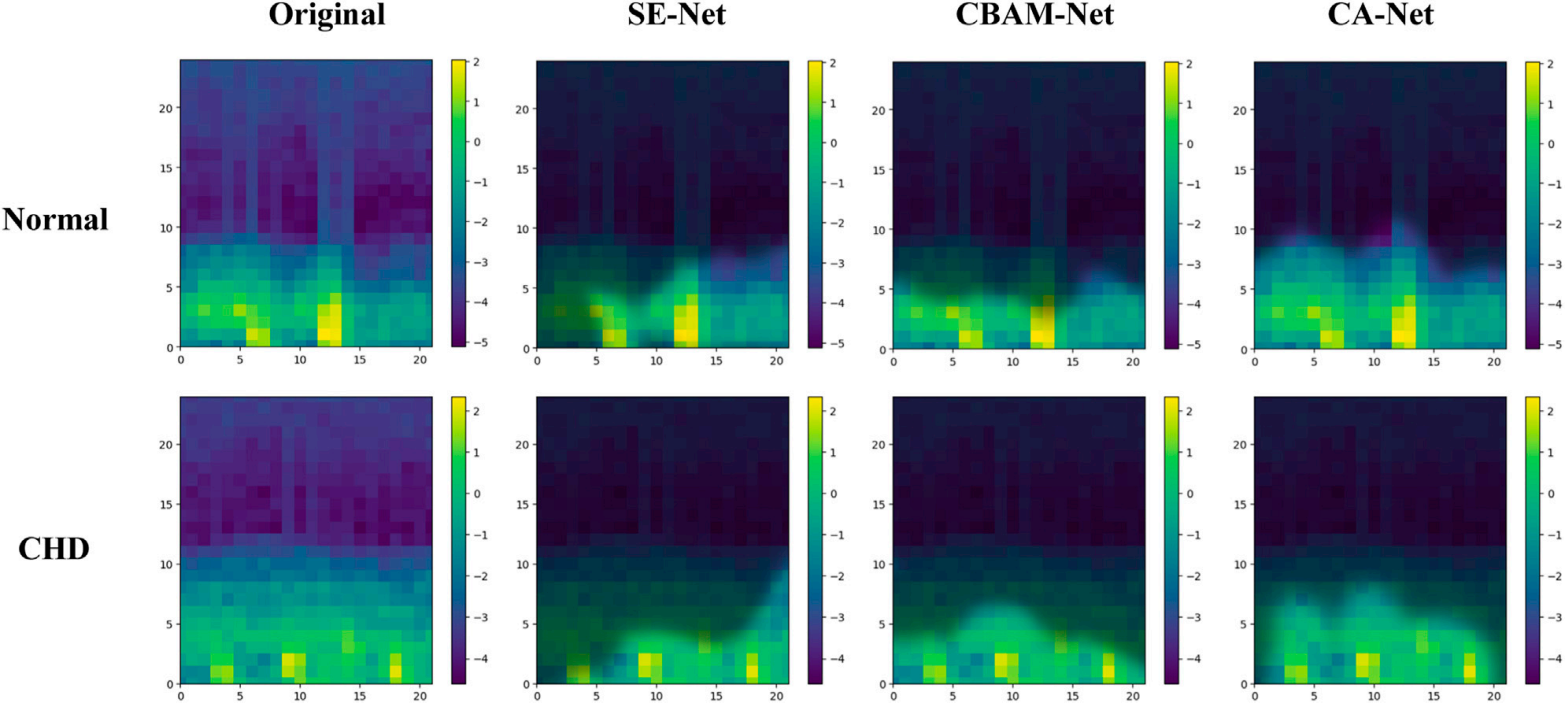
Attended regions under different attention mechanisms.

The effectiveness of incorporating attention mechanisms will be analyzed in this section, considering both experimental theory and results. Within the field of computer vision, attention mechanisms find widespread use due to the constraints posed by visual scope and information processing. They enable the extraction of valuable information while disregarding data that may be irrelevant or unnecessary. By integrating attention mechanisms into neural networks, the network’s focus can be directed toward salient feature regions during the analysis. In this study, three types of attention mechanisms were introduced for comparative experiments, to enhance the concentration on the feature information of S1 and S2. Visualized in Figure 3, the impact of different attention mechanisms on the S1 and S2 features within the feature maps is depicted, highlighting the attended regions. While the network structure remains consistent apart from the attention mechanism modules, it is evident from the results that Channel Attention (CA) surpasses other attention mechanisms in its ability to identify pathological regions, as illustrated in Figure 8. The focusing effect of CA is further supported by the evaluation metrics presented in Tables 4, 5.

### 4.3 Analysis of comparative ablation experiments

Clinical research has revealed that regions implementing early screening for congenital heart disease (CHD) have demonstrated notably elevated rates of treatment success in contrast to areas lacking such screening protocols. The prevailing strategy for CHD screening predominantly hinges on the expertise of physicians in the field of auscultation. Nevertheless, this approach is characterized by inefficiency, susceptibility to diagnostic errors, and a substantial reliance on clinical experience. As a result, the primary objective of our study is to leverage automated analysis of cardiac sounds to offer supplementary diagnostic support for CHD, thereby augmenting both the precision and efficiency of the screening process.

To establish the credibility of the classification model, we undertook comparative experiments against the latest developments in the field and conducted ablation studies. About the work by the Rizal research team(Rizal et al., 2022), commendable classification outcomes were attained on a limited dataset through the use of conventional Short-Time Fourier Transform (STFT) techniques. The Nehary research team, on the other hand, introduced Mel-Frequency Cepstral Coefficients (MFCC) (Nehary et al., 2021)—frequently employed in speech recognition—and Mel-Frequency Spectral Coefficients (MFSC), which implement triangular filter banks resembling the human auditory system. The feature extraction process of MFCC is enhanced by the incorporation of Discrete Cosine Transform (DCT). Within the context of ablation experiments, we juxtaposed single MFSC features with envelope features for feature selection. In the architecture of the classification backbone network, we contrasted models that lacked supplementary attention mechanisms against those augmented with Squeeze-and-Excitation (SE) modules, as well as Convolutional Block Attention Module (CBAM) modules.

Several feature extraction and classification methods achieved favorable results as shown in the tables above. However, the LCACNN model based on fusion features was significantly better than the other methods. The results showed that:

(1) In comparative ablation experiments, our classification model showed superior performance in three evaluation metrics: Accuracy (Acc), Modified Accuracy (MAcc), and F-score. Higher Acc values indicate that the local superposition fusion is better at highlighting the features of the S1 and S2 heart sounds, thus improving the detection rate of patients during screening. Higher MAcc values indicate that the fusion features have a higher resistance to noise. Higher F-scores indicate that our classification model produces fewer false negatives.
(2) In both comparative and ablation experiments, the Sensitivity (Se) value of our classification model was notably higher than other algorithm models, affirming its lower misdiagnosis rate for CHD diagnosis.
(3) In the PhysioNet database, MFSC combined with a CNN model showed slightly higher Specificity (Sp) values than our algorithm, but significantly lower Se values, indicating an increase in false negatives. Such a risk is unacceptable for patients.
(4) In the ablation experiments, our algorithm outperformed the SE and CBAM modules on the comprehensive MAcc and F-score evaluation metrics, confirming the effectiveness of the Channel Attention (CA) mechanism in focusing on characteristic CHD regions.
(5) The various parameter indicators of our classification model were verified using the PhysioNet database and the HS database, rendering the experimental data more reliable.

### 4.4 Limitations and future work of this study

Two limitations are observed in this study. Firstly, Atrial Septal Defect (ASD), Ventricular Septal Defect (VSD), and Patent Ductus Arteriosus (PDA) constitute common types of congenital heart disease (CHD). However, the requirement for screening solely entails determining disease presence. Consequently, multi-class experiments were not conducted in this study. Further research could potentially differentiate among these specific diseases. Secondly, high-risk areas for CHD include remote mountainous regions. Yet, these regions often encounter poor network connectivity, potentially impacting the diagnosis of the cloud-deployed cardiac sound analysis model. Thus, future research might contemplate the utilization of lighter models, embedded within edge computing devices, to enhance diagnostic efficiency. Despite these limitations, the automated cardiac sound analysis models still retain the potential for significant application in clinical screening, contributing to the conservation of medical resources and cost reduction.

## 5 Conclusion

We have developed and validated the LCACNN cardiac sound automatic classification model based on fusion features. This model is employed for assisting in the diagnosis between normal and patient cases, thereby enhancing patients’ survival rates and holding significant implications for early screening of congenital heart disease.

## Data Availability

The raw data supporting the conclusion of this article will be made available by the authors, without undue reservation.
